# Exploring the Sociodemographic and Clinical Features of Extrapulmonary Tuberculosis in Saudi Arabia

**DOI:** 10.1371/journal.pone.0101667

**Published:** 2015-02-03

**Authors:** Sahal Al-Hajoj, Mohammed Shoukri, Ziad Memish, Raafat AlHakeem, Fahad AlRabiah, Bright Varghese

**Affiliations:** 1 Department of Infection and Immunity, King Faisal Specialist Hospital and Research Centre, Riyadh, Saudi Arabia; 2 National Biotechnology Centre, King Faisal Specialist Hospital and Research Centre, Riyadh, Saudi Arabia; 3 Preventive Medicine Directorate, Ministry of Health, Riyadh, Saudi Arabia; 4 Department of Medicine, King Faisal Specialist Hospital and Research Centre, Riyadh, Saudi Arabia; University of Hyderabad, INDIA

## Abstract

**Background:**

Saudi Arabia annually reports a relatively higher proportion (28–32%) of extrapulmonary tuberculosis (EPTB) cases in comparison to other global regions. However, there were few studies conducted so far to determine the sociodemographic factors and clinical manifestations associated with EPTB at a nationwide level.

**Methodology:**

A retrospective analysis on culture positive EPTB isolates collected from all the provinces of the country were conducted for a period of 12 months to determine the spectrum of diversity in EPTB infection sites and the confounding factors. A detailed clinical and demographical data analysis was carried out along with first line drug susceptibility testing.

**Principal Findings:**

Intra-thoracic and extra-thoracic lymph nodes (44.6%) were the most common sites of infection followed by gastrointestinal (17.3%) and central nervous systems (11.8%). Male patients were mostly infected (58.8%), in contrary to the global trend. Any drug resistance was observed in 23.1% isolates with a 2.1% of multi-drug resistance. HIV reactivity was found only in 2.2% cases. A higher proportion of Saudi nationals (58.8%) were infected compared to the immigrants, descending mostly from South Asia (34.4%) and South East Asia (31.2%). The Saudi population predominated with all forms of EPTB while immigrants showed no significant variations.

**Conclusions:**

Saudi Arabia faces a serious threat from EPTB, particularly to the central nervous system and gastrointestinal systems. More effective diagnostic strategies and control measures must be implemented to reduce the high rate of EPTB in the country. In addition, these findings warrant further detailed research to explore all related comorbid conditions of EPTB development, particularly the host-related factors.

## Introduction

Tuberculosis (TB) remains a major public health problem with 8.6 million new incident cases including 0.8 million extra pulmonary tuberculosis (EPTB) cases and 1.3 million deaths globally [1]. A decreasing or stable trend of new TB cases was observed in developed or industrialized nations. However, some developing countries show an increasing trend, whereas many others exhibit a very slow decline in incidence [1]. The deadly synergisms between TB and HIV with the emergence of drug resistance have complicated the control of TB [1]. Generally, a rising proportion of EPTB has been reported from different countries [1–3]. Furthermore, the HIV epidemic and rising numbers of immigrants from developing countries to developed countries are assumed to be responsible for the changes in this elevation in many regions [1,4,5]. Other immunosuppressive conditions also tend to maximize the frequency of EPTB [6,7]. Younger age, female gender, African and Asian origin also proved to be risk factors in different studies [8–10]. EPTB receives less interest than pulmonary tuberculosis because of its low infectious potential. However, it is considered a serious clinical problem because of the diagnostic challenges encountered and the propensity to cause high morbidity and mortality [4,5]. A recent study from Brazil also highlighted the lack of diagnostic capabilities as a major reason for late diagnosis and management of EPTB [11].

Saudi Arabia consistently reports an increasing trend of EPTB (29.3% in 2006 compared to 31.8% in 2011) [12]. According to WHO statistics, the Eastern Mediterranean region (which includes Saudi Arabia) reports the highest rate of EPTB (22%) globally [1,12,13]. The Saudi population showed higher proportions of EPTB compared to migrant populations, which accounted for >50% of annually reported cases (59.7% and 55.9% respectively in 2009 and 2010). During the same period, pulmonary TB was also reported with an equal distribution among both Saudi (51.45 and 49.7% respectively) and non-Saudi populations (48.5% and 50.3% respectively) [12,13]. Interestingly, several retrospective analyses from different institutions in Saudi Arabia showed a huge diversity of clinical manifestations of EPTB [14,15]. Limited nationwide studies, which only analysed the culture confirmed EPTB case data comprehensively exploring the various clinical presentations with its distribution according to the sociodemographic factors. Therefore, this study has been designed to review the sociodemographic and clinical features of EPTB cases for a period of 12 months utilizing nationwide collection of culture positive subjects. As a cross sectional analysis, this study investigated the demographic composition or characteristics of the study population and their relationship to the development of EPTB in the country.

## Materials and Methods

### Study subjects

A nationwide collection of culture positive *M*. *tuberculosis* isolates was carried out in all the provinces of the country, as part of a national surveillance study between June 2009 and July 2010. The objectives of this current study were not part of the previously published main study [16]. Patient’s clinical and demographical data were collected using standard data collection forms and further verified with the national TB registry maintained by the Ministry of Health. Patients in whom the site of infection was confined to lungs were considered as pulmonary TB and infection extended to other organs or tissues outside lungs were considered as extrapulmonary involvement. Patients who had both pulmonary and extrapulmonary involvements were excluded from the analysis of EPTB based on WHO sample selection policy [1]. The study has been reviewed and approved by the research ethical committee of King Faisal Specialist Hospital and Research Centre, Riyadh, Saudi Arabia. All the data collected for the study was anonymized and no patient identifiers were used throughout the data collection and analysis period. As the study utilised data from the national TB registry (an anonymous case registry) only, the need for informed consent was waived.

### Study design and methods

All the isolates were subjected to identification with the commercial line probe assay-Genotype Mycobacterium MTBC (Hain Lifescience, Nehren, Germany) as *M*. *tuberculosis* complex following manufacturer’s instructions. All the extrapulmonary isolates collected during the study period were subjected to first line drug susceptibility testing by using the commercial kit MGIT SIRE (Becton Dickinson, MD, USA). The following drug concentrations were tested: 1 μg/ml for streptomycin,

0.1 μg/ml for isoniazid, 1μg/ml for rifampin, and 5 μg/ml for ethambutol.

### Data analysis

The study group was divided into “Saudi” (SA-people who hold Saudi nationality) and “Non-Saudi” (NSA-people only with a resident permit and hold other nationalities) for detailed analysis. The NSA groups were further sub-divided into South Asian (SAS), South East Asians (SEA), Africans (AFR), and Arabs (ARB) according to the geographical area of their origin.

The study group SAS consisted of people from India, Pakistan, Bangladesh, Myanmar, Nepal, Sri Lanka and Afghanistan. The study group SEA consisted of people from the Philippines and Indonesia. The study group ARB consisted of people from Yemen, Lebanon, Syria and Egypt. The AFR group consisted of people from Sudan, Eritrea, Ethiopia, Djibouti, Chad, Nigeria and Somalia.

The sites of infection were divided into six major groups; lymph nodes (intra-thoracic and extra-thoracic), gastrointestinal system, central nervous system, osteoarticular, pleural and urogenital. All other sites of infection were considered as part of a seventh group identified as “other rare forms” for convenient statistical analysis. All statistical analyses were carried out by using the software package SPSS version-20 (IBM Corporation, NY, USA).

## Results

During the study period, 381 extrapulmonary isolates were enrolled from all nine provinces of the country. Higher proportions of male patients (57.5%) were observed compared to the female population. The age groups of the enrolled cases showed a predominance of young patients (16–29 years-37.5% and 30–44 years-33.9%). The nationality of the patients clearly showed a higher number of Saudi nationals (58.8%). However, sub-classification of non-Saudi population demonstrated majority of people belonged to SAS (34.4%) followed by SEA (31.2%), AFR (20.4%) and ARB (14%), respectively. Of the 381, 195 (51.2%) were AFB smear positive. HIV reactivity testing results were only available for 48.8% of cases and 4 (2.1%) cases were found to be reactive. Overall drug resistance rates among the study isolates were 23.1%. Mono drug resistance was noticed in 64 (16.8%) cases while poly-drug resistance was 4.2%. Multi Drug Resistant TB (MDRTB) rates observed in the study were only 2.1% (Table 1).

**Table 1:** Demographical and clinical summary of 381 EPTB cases.

**Parameters**	**Cases/percentage**
Gender	
Male	219(57.5)
Female	162(42.5)
**Age groups**	
Up to 15	33(8.6)
16–29	143(37.5)
30–44	129(33.9)
45–59	49(12.9)
>60	27(7.1)
Nationality	
Saudi	224(58.8)
Non-Saudi	157(41.2)
-ISC^1^	54(34.4)
-SEA^2^	49(31.2)
-AFR^3^	32(20.4)
-ARB^4^	22(14.0)
**Smear**	
Positive	195(51.2)
Negative	186(48.8)
**HIV**	
Positive	4(2.1)
Negative	182(97.8)
**Drug Resistance**	
Mono Drug Resistance	64(16.8)
Streptomycin	31(48.4)
Isoniazid	27(42.2)
Rifampicin	3(4.7)
Ethambutol	3(4.7)
Poly-drug Resistance	16(4.2)
MDR-TB^5^	8(2.1)
Pan Susceptible	293(76.9)

^1^ Indian sub-continent-India, Pakistan, Bangladesh, Myanmar, Nepal, Sri Lanka.

^2^ South East Asia-Philippines and Indonesia

^3^ Africa-Chad, Cameroon, Djibouti, Eritrea, Nigeria, Ethiopia, Somalia, Sudan

^4^ Arab countries-Egypt, Syria, Yemen, Lebanon

^5^ Multi-drug resistant tuberculosis

**Figure 1:**
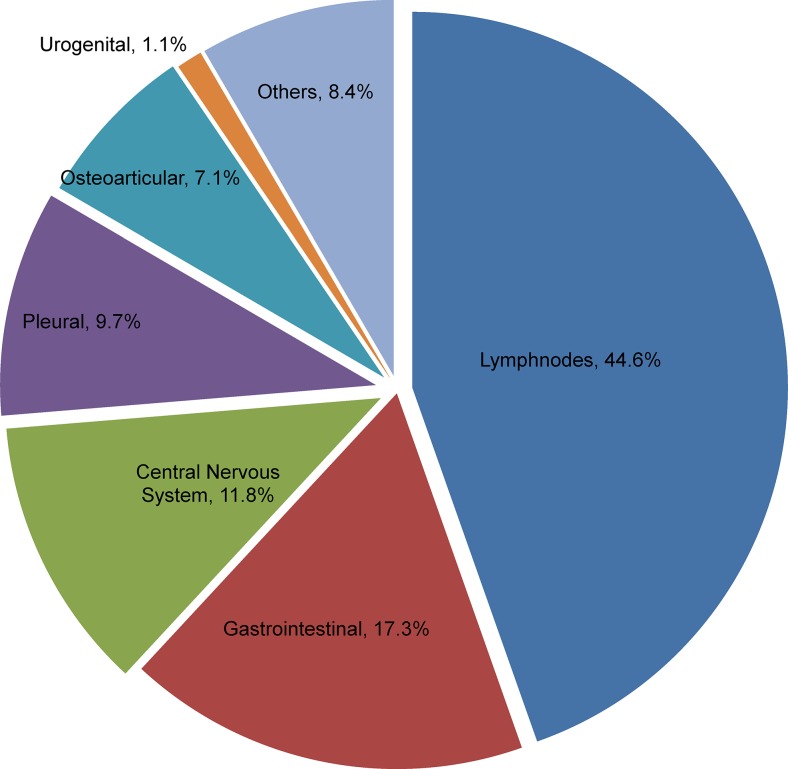
Distribution pattern of extra-pulmonary infection sites among 381 patients during 2009–2010. Figure shows the proportions of different extrapulmonary sites of infections reported among the culture positive cases during the study period.

**Figure 2:**
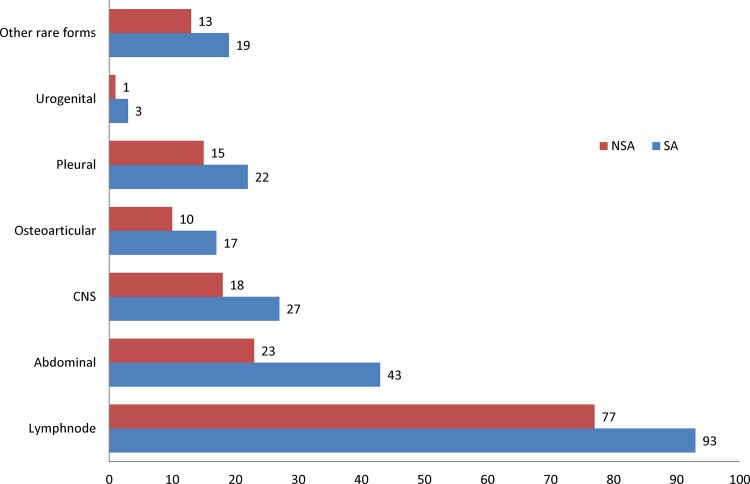
Major extra-pulmonary infection sites among local and immigrant populations of Saudi Arabia. Distribution of various extrapulmonary sites among Saudi Arabian and Non-Saudi Arabian population.

The most predominant site of infection in the study was lymph nodes (44.6%) followed by gastrointestinal (17.3%), central nervous system (11.8%) and pleura (9.7%) (Fig. 1). An analysis on nationality of the patients with reference to the site of infection showed a distinct higher representation of Saudi population. Generally all the sites of infection were predominant among the Saudi population compared to the immigrant population. However, gastrointestinal, urogenital and central nervous system sites demonstrated a higher propensity among Saudi population while other sites were observed with a nominal increment in distribution (Fig. 2). Lymphatic TB was reported as high among female patients (55.9%), while male patients showed a predominance of gastrointestinal TB (80.3%). All other sites of infection were also found to be relatively high in number among male patients (Table 2). Gastrointestinal (36.4%), central nervous system (44.4%), pleural (32.4%), urogenital (75%) and other rare forms (65.6%) of EPTB were largely observed within the 30–44 year age group. However, the group aged 16–29 showed higher proportions of lymph node (45.9%) and osteoarticular TB (44.4%). The group aged <15 years showed a higher occurrence of lymph node TB (54.5%) (Table 2).

**Table 2:** Proportions of EPTB site diversity based on demographical data.

	Lymphnodes	GIT^1^	CNS^2^	Pleural	OA^3^	UG^4^	Others^5^
Total cases	170(44.6%)	66(17.3%)	45(11.8%)	37(9.7%)	27(7.1%)	4(1.1%)	32(8.4%)
Gender							
Male	75	53	29	24	16	3	19
Female	95	13	16	13	11	1	13
Age groups							
Up to 15	18	6	5	2	1		1
16–29	78	22	13	11	12	1	5
30–44	44	24	20	12	6	3	21
45–59	24	8	4	6	3	0	4
>60	6	6	3	6	5	0	1
Origin^**6**^							
Saudi	93	43	27	22	17	3	19
SAS	22	7	8	9	2	-	6
SEA	33	5	3	2	2	-	4
AFR	11	9	3	3	4	1	1
ARB	11	2	4	1	2	-	2

^1^ Gastro Intestinal Tract

^2^ Central Nervous System

^3^ Osteoarticular

^4^ Urogenital

^5^ Pericardial, Kidney, Skin, Miliary, Disseminated, abscesses other than classified sites.

^6^ SAS-South Asian, SEA-South East Asian, AFR-African, ARB-Arab

## Discussion

A cross-sectional analysis on sociodemographic factors in relation to the development of EPTB as well as the diversity of infection sites have been studied on a nationwide scale or coverage. Increasing incidence of EPTB, even with the reported low proportions of classical co-morbid factors for TB (ie; HIV, alcohol or drug abuse, homelessness) in the country, makes the findings more relevant to be discussed.

The EPTB was dominant among the groups aged, 16–29 and 30–44 years, which exactly follows the trend of higher occurrence of TB incidences in the young people [12]. The group aged >60 years were the least affected by EPTB in this study. Similar observations have been made in different parts of the world on large scale studies [8,17,18]. The children (aged <15 years) mainly showed a high incidence of lymphnode TB. This might probably be related to the recent outbreaks of BCG vaccine-associated lymphadenitis reported from different regions in the country [19]. The middle aged patients (30–44 years) were reported with EPTB in all sites, which is in concordance to previous studies which identified middle age as a major risk factor for EPTB [5,9].

The gender of patients showed a vastly different outcome against the trend in other global regions as in this study we observed a higher rate of EPTB in male patients. Similar trends of male gender predominance were constantly reported among the EPTB patients by the Ministry of Health in Saudi Arabia since 2005 [6,12,13]. This is an opposite finding compared to the Western world, where EPTB rates are relatively high among females [5,10].

Analysis of geographic origins of the patients showed the predominance of Asians particularly from the SAS region followed by SEA. This is an interesting finding as results were similarly observed in developed nations. A study from the USA clearly showed a predominance of South Asians among the largely reported extrapulmonary cases for a period of 12 years [4]. South East Asians and Africans were two major patient groups with higher rates of EPTB in the current study. However, the Arabs were reported with the lowest incidence of EPTB. These variations in the EPTB rate may relate to the rate of TB prevalence reported in countries from where the patients originated. The countries in these regions reported an annual prevalence of 109 to 489 cases/100,000 according to recent WHO reports [1]. In addition, the higher rate of reactivation of latent TB among immigrants in Saudi Arabia was also reported recently [20]. Current study findings conform with previous reports that reactivated TB among immigrants are more EPTB than pulmonary [8]. Thus, we assume Saudi Arabia also follows the same trend of TB reactivation with higher proportion of EPTB, even though further detailed research is needed.

The most frequent form of EPTB in Saudi Arabia was TB of various lymph nodes (extra-thoracic and intra-thoracic) and this finding is similar to other global regions [2,4,8,9,21,22]. However, the most striking infection sites in the country were gastrointestinal and central nervous system. This finding is in opposition to other parts of world as the distribution of infection sites varies from one region to another [2,4,8,9,23]. The implications of higher prevalence of these two sites which have been reported rare in some other parts of the world should be monitored closely in the country. To explain, in USA, according to the recent statistics of the CDC, the proportions of different sites of infection shows mainly lymphatic system involvement followed by pleural, osteoarticular and peritoneal [3]. On the other hand, in the European Union, a report from 11 countries showed the same trend of higher prevalence of lymphatic TB. Pleural TB existed as the second most common form followed by central nervous system and osteoarticular TB in most of the countries [23]. A recent Saudi Arabian study that was conducted in 2010–2011on nationwide coverage also reported high prevalence of lymphatic TB followed by bone, nervous and gastrointestinal systems among the studied population [24], a finding slightly deviating from this present study. However, the difference in representation of infection sites in both studies may be mostly related to the sample selection strategy used as the current study was based only on culture positive cases. Data was scarce on the site specific distribution of EPTB in Asian and African countries. Retrospective studies from Nepal and Pakistan showed similar trends with Saudi Arabia with predominance of gastrointestinal and central nervous system TB after lymphatic TB [21,25].

The remaining three groups-urogenital, pleural and other rare forms were also reported with considerable proportion. The other rare forms included TB of the pericardium, breast, skin, kidney, miliary and disseminated. The proportion of other rare sites of infection (8.4%) in the current study is higher than that of the Netherlands or Poland but similar to many other countries in Europe and America [18,22,23]. Furthermore, the importance of all forms of EPTB in the developing countries has not yet been ascertained due to lack of diagnostic facilities and difficulties in diagnosis. The constant likelihood of Saudi populations with EPTB compared to the immigrant population in the recent years particularly with domination of central nervous system and gastrointestinal TB is a noticeable concern. Perhaps both forms of TB are rare in distribution in the developed world. However, only a few developing countries demonstrated the same trend of central nervous system and gastrointestinal TB predominance [21,25]. The High incidence of gastrointestinal TB among the Saudi population (65.2%) compared to the immigrant population is a new finding which needs further comprehensive analysis to conclude the possible reasons for this higher occurrence.

Furthermore, we assume that the higher proportion of EPTB among the Saudi population may have a direct correlation with the host-related factors, mainly with the larger amount of immunosuppressive illnesses common in the country. A retrospective study reported neoplasia/ immunosuppression, diabetes mellitus, chronic use of steroids and HIV as serious confounding factors among EPTB patients in Saudi Arabia [15]. The role of primary immunodeficiencies (PID) to make the host susceptible to tuberculosis was clearly observed in different studies [26–28]. Saudi Arabia reported the highest incidence (up to 67%) of consanguinity and related immune disorders in the world, which may have a lot of implications that need to be explored [29,30]. Moreover, the increased rate of several PIDs such as chronic granulomatous disease (5.2 cases/100,000 live births), severe combined immunodeficiency syndrome (19 cases/100,000 live births) are the highest in the world among Saudi population [31,32]. The consequences of PIDs on various infectious diseases including tuberculosis development have been reported previously from different regions including Saudi Arabia [33,34]. Thus, we insist on the fact that the large existence of PIDs in the country may have a direct influence on the large scale consistent reporting of EPTB predominance among Saudi Arabian populations. HIV which is the most common risk factor for TB has not been reported in large levels in the country as the incidence of HIV still remains <4 cases/100,000 populations annually [35]. In addition, other co-morbid factors such as, metabolic syndromes, vitamin-D deficiency and various genetic polymorphisms, have also been proved risk factors for EPTB. Existence or higher prevalence of these factors was also reported from Saudi Arabia [36,37].

The drug resistance in the study followed the same course of overall resistance reported in the previous study [16]. The overall resistance in the previous study was 23.6% and pulmonary TB reported with a rate of 23.8% resistant isolates. In relation with the previous study, the drug resistance in the EPTB isolates was found to be 23.1%. The MDRTB rate was also comparatively low among the EPTB isolates (2.1% EPTB and 4.5% pulmonary TB isolates) [16].

This study has certain limitations; the sampling was limited only to culture positive EPTB cases alone for a 12-month period only and a detailed clinical history of the patients were not collected to analyse the risk factors. The date of arrival of the non-Saudi patients to the country was not available to rule out the time span of disease activation and a further follow-up of the cases was not conducted.

## Conclusions

EPTB is a serious concern to public health in Saudi Arabia and it mainly infects Saudi nationals, which is in contrary to the trend of pulmonary TB (higher incidence among immigrants). The marginal increase in their incidence rates and proportions of the sites of infection show a unique trend compared to developed countries. In addition, the contradictory findings compared to other countries and relatively higher occurrence of certain sites of infections (ie. gastrointestinal- 65.2%) among Saudi population need further verification to find the host-related risk factors. Thus, the current findings warrant a nationwide study to determine the related risk factors for EPTB development in Saudi Arabia.
